# Microalgae and Phototrophic Purple Bacteria for Nutrient Recovery From Agri-Industrial Effluents: Influences on Plant Growth, Rhizosphere Bacteria, and Putative Carbon- and Nitrogen-Cycling Genes

**DOI:** 10.3389/fpls.2019.01193

**Published:** 2019-09-27

**Authors:** Somayeh Zarezadeh, Navid R. Moheimani, Sasha N. Jenkins, Tim Hülsen, Hossein Riahi, Bede S. Mickan

**Affiliations:** ^1^Faculty of Life Sciences and Biotechnology, Shahid Beheshti University, Tehran, Iran; ^2^Algae R and D Centre, Murdoch University, Perth, WA, Australia; ^3^Centre for Sustainable Aquatic Ecosystems, Harry Butler Institute, Murdoch University, Perth, WA, Australia; ^4^UWA School of Agriculture and Environment (M079), The University of Western Australia, Perth, WA, Australia; ^5^The UWA Institute of Agriculture (M082), The University of Western Australia, Perth, WA, Australia; ^6^Advanced Water Management Centre, The University of Queensland, Brisbane, QLD, Australia; ^7^Richgro Garden Products, Jandakot, WA, Australia

**Keywords:** microalgae, purple phototrophic bacteria, organic fertilizer, Lolium rigidum, rhizosphere bacteria, PICRUSt

## Abstract

Microalgae (MA) and purple phototrophic bacteria (PPB) have the ability to remove and recover nutrients from digestate (anaerobic digestion effluent) and pre-settled pig manure that can be Utilized as bio-fertilizer and organic fertilizer. The objective of this study was to compare the effectiveness of MA and PPB as organic fertilizers and soil conditioners in relation to plant growth and the soil biological processes involved in nitrogen (N) and carbon (C) cycling. To this end, a glasshouse experiment was conducted using MA and PPB as bio-fertilizers to grow a common pasture ryegrass (*Lolium rigidum* Gaudin) with two destructive harvests (45 and 60 days after emergence). To evaluate the rhizosphere bacterial community, we used barcoded PCR-amplified bacterial 16S rRNA genes for paired-end sequencing on the Illumina Mi-Seq. Additionally, we used phylogenetic investigation of communities by reconstruction of unobserved states (PICRUSt) analysis for the detection of putative functional genes associated with N and soil-C cycling. There was a significant increase in plant growth when the soil was amended with PPB, which almost performed as well as the chemical fertilizers. Analysis of the rhizosphere bacteria after the second harvest revealed a greater abundance of Firmicutes than in the first harvest. Members of this phylum have been identified as a biostimulant for plant growth. In contrast, the MA released nutrients more slowly and had a profound effect on N cycling by modulating N mineralization and N retention pathways. Thus, MA could be developed as a slow-release fertilizer with better N retention, which could improve crop performance and soil function, despite nutrient losses from leaching, runoff, and atmospheric emissions. These data indicate that biologically recovered nutrients from waste resources can be effective as a fertilizer, resulting in enhanced C- and N-cycling capacities in the rhizosphere.

## Introduction

With the global population expected to reach between 6.9 and 12.6 billion by 2100 (Kc and Lutz, 2017), there is increasing pressure on agricultural production to provide food, which, in turn, has led to the intensification of fertilizer use. Consequently, large quantities of chemical fertilizers are applied to agricultural soils to maintain crop productivity and profitability. In the last decades of the twentieth century, the application of these fertilizers has led to twofold increases in food production (Tilman, 1999). However, this expansion in agriculture and the excessive use of chemical fertilizers have adversely affected the environment, including nitrogen (N) and phosphorus (P) loss *via* runoff, leaching, and volatilization (more than 70% of N is lost this way), leading to the eutrophication of aquatic systems (Carpenter, 2005; Matassa et al., 2015), increased greenhouse gas emissions (Shcherbak et al., 2014), and decreased soil fertility (e.g., because of acidification and reduced water storage capacity; see Gyaneshwar et al., 2002). Thus, there is an urgent need for more sustainable N fertilizer use without compromising crop performance. Consequently, farmers, ecologists, and consumers, particularly millennials, are reconsidering the use of organic fertilizers (Chang et al., 2007).

The application of organic fertilizers has many benefits for soil, including improved soil structure, water retention, pH levels, and disease suppression, as well as providing macronutrients and micronutrients for plant growth, as has been recently reviewed (Abbott et al., 2018). The activity of enzymes, such as dehydrogenase, protease, urease, cellulase, β-glucosidase, and phosphatase, in soils amended with organic fertilizers is typically higher than it is in soils treated with chemical fertilizers (Chang et al., 2007). Organic amendments to soil can also increase the soil’s microbial biomass (Pascual et al., 2000; Peacock et al., 2001; Chang et al., 2007) and influence the composition and function of the soil’s microbial community (Peacock et al., 2001; Mickan et al., 2018). Soil microorganisms are important to soil function because they participate in the formation of soil structure, mineralize soil organic matter (SOM), and recycle N and C (Pascual et al., 2000; Zhong et al., 2010; Mickan et al., 2018)—all of which make these nutrients available to plants (Peacock et al., 2001).

While the main components of chemical fertilizers are produced *via* Haber–Bosch (N) and from the mining of phosphate rock and potash, organic fertilizers are produced *via* composting and the recycling of livestock waste, such as poultry, cow, and pig manure. Other potential sources for organic fertilizer production include domestic and agri-industrial wastewaters, which contain varying levels of organics, N, P, and potassium (among other constituents) that can be partly recovered from biological sludge. In fact, it has been estimated that global phosphorus (Cordell et al., 2009) and potassium demands could largely be serviced from waste streams (human and animal), while minimizing geological inputs; further, up to 50% of the global N market could be supplied using this method (Batstone et al., 2015), thereby reducing the anthropogenic N load of Haber–Bosch by around 50 Mt per annum.

However, to realize and maximize the recovery of resources from wastewater, novel treatment technologies have to be applied. Conventional methods, such as the activated sludge process or biological nutrient removal, have been designed to remove rather than recover, which results in the dissipation of C as CO_2_ (∼50%), N as N_2_ (∼90%), and phosphorus as metal bound sludge (as a result of Al or Fe precipitation). While conventional technologies have been applied for more than 100 years (Ardern and Lockett, 1914) and are fit for purpose, the current paradigm shift in wastewater treatment focuses with increasing intensity on the recovery and upgrade of resources from wastewater.

In this context, new platforms have been proposed to realize this shift (Verstraete et al., 2009; McCarty et al., 2011), and photosynthetic and phototrophic organisms, such as microalgae (MA) and purple phototrophic bacteria (PPB), have the potential to play a major role in this development. Both MA and PPB have been reported to effectively partition carbon (C) and nutrients (N and P) from the soluble into the solid phase (Hülsen et al., 2014; Marazzi et al., 2017) and can act as a mediator for the biological up-concentration of nutrients in a concentrated, low-volume stream. One of the main features is high biomass yields (close to unity), as both MA and PPB generate energy (ATP) from light while effectively assimilating C *via* photoautotrophic growth with UV-VIS (400–700 nm) (MA) or photoheterotrophic growth (PPB) with infrared (>800 nm). High biomass yields result in high resource recovery, which increases the potential of recycling substantially.

Bulk MA and PPB biomasses have been applied as feed and feed additives, as anaerobic digestion substrates for energy production, as resources for biofuels, and as organic fertilizers (Chew et al., 2017; Dahiya et al., 2018). Using MA and PPB biomasses as organic fertilizers has received specific attention over the last two decades (Harada et al., 2005; Mulbry et al., 2005; Wu et al., 2013; Coppens et al., 2016; Wuang et al., 2016; Schreiber et al., 2018). Recently, the effects of wet and dried MA biomasses on the growth of wheat have been reported to be comparable to chemical fertilizers when balanced for P (Schreiber et al., 2018). Additionally, there is a growing body of evidence that different kinds of MA and cyanobacteria could affect the growth of different crops (Shariatmadari et al., 2015; Barone et al., 2017). Generally, the application of wastewater-derived MA biomasses as an organic, slow-release fertilizer enhances plant growth rates (Mulbry et al., 2005; Coppens et al., 2016; Wuang et al., 2016). Similar effects have also been described for PPB, for which stimulatory effects on the grain yield of rice (Harada et al., 2005), on the growth of stevioside (Wu et al., 2013), and on the germination, growth, and lycopene content of tomatoes (Lee et al., 2008) have been reported. It is also commonly assumed that PPB can act as a plant growth–promoting rhizobacteria, enhancing plant growth by increasing the activity of soil microorganisms, producing phytohormones, and facilitating nutrient uptake (Harada et al., 2005; Liang et al., 2010; Wong et al., 2014). Similarly, when used as an organic fertilizer, algae have been shown to be a biostimulant for soil microorganisms, the activity of which affects plant growth. However, the mechanisms, modes of action, and microorganisms involved in this process are not fully understood.

The addition of organic C to soil in the form of organic matter provides a source of energy, C, and nutrients to microbial communities (Sun et al., 2015). This also influences the bacterial composition and function—in particular, the taxa involved in C transformation and N cycling (Mickan et al., 2018). To date, there is no information about the effects of MA and PPB grown on waste and used as organic fertilizer on the bacterial community of soil in relation to plant growth. For the current study, we investigated the effects that dried MA cultivated on anaerobic digestate and dried PPB cultivated on piggery effluent have on the microbial community of soil, how these microorganisms decompose organic matter into inorganic matter, and how they affect the nutrient cycles of the soil.

We hypothesized that:

- The application of dried MA and PPB as sources of organic N would stimulate soil N mineralization and result in plant growth promotion.
- These organic fertilizers would also increase the relative abundance of bacteria involved in N transformation pathways, especially nitrification, resulting in an improved nutrient status for the soil.
- The application of dried MA and PPB as sources of SOM and available C would increase the abundance of fast-growing copiotrophic bacteria and genes involved in C degradation.

## Materials and Methods

### The Cultivation and Harvesting of MA and PPB

#### The Growth and Harvesting of MA

The MA fertilizer is a mixed culture of *Chlorella* sp. and *Scenedesmus* sp., which was cultivated in a 1 m^2^, 0.1 m deep raceway pond under outdoor conditions at Murdoch University’s Algae Research and Development Centre with a constant ammonium concentration of 100 mg $NH_{4}^{+}-N\;L^{-1}$. The culture was grown on a food waste–derived anaerobic digestion digestate that was filtered (<1 mm) to remove solids. The culture’s average algal biomass productivity was 10 g m^−2^ day^−1^. The harvested MA were oven-dried at 60 degrees Celsius for 3 days and then ground to particles of less than 1 mm and stored at 5 degrees Celsius until the experiment was potted. The composition of dried MA is shown in **Supplementary Table S1**.

#### The Growth and Harvesting of PPB

The PPB were grown on indoor pig farm wastewater. The wastewater was taken from the growers’ shed and contained feces, urine, wash water, undigested feed, and other gritty particulates (e.g., pig hair and sand). Before feeding the outdoor PPB growth system, the wastewater was settled for 30 min in a 1,000 L IBC container. PPB biomass, with a composition of up to 57% *Rhodopseudomonas* sp., was grown in three flat plate reactors (100, 80, and 60 L in volume). Each reactor was filled with pre-settled wastewater and continuously mixed for 5 days. The flat plate reactors were designed to favor the attached biofilm formation of PPB on the reactor walls. After draining the reactors, the attached PPB biofilm was harvested at around 10% dry solids *via* scraping from the reactor walls. The harvested biomass was transported on ice (1 h) and then stored in a freezer. The following analyses were carried out to determine the wet biomass composition: total solids, volatile solids, chemical oxygen demand, total Kjeldahl N, total phosphorous, ammonium (NH_4_-N), phosphate (PO_4_-P), trace metals, and amino acids. The analytical methods used are detailed in Hülsen et al. (2018). The dried PPB composition is shown in **Supplementary Table S1**.

### Soil Collection and Analysis

The top 10 cm of dry soil from fields at Murdoch University (32° 04′ 23″ S, 115° 50′ 18″ E) was collected on 11 May 2018. The gathered soil was sieved with a 2 mm mesh sieve and then mixed completely. The pot capacity was determined following Ogbagaa et al. (2014). The soil-to-water ratio of 1:5 was used to measure the soil’s pH level and conductivity. For this purpose, the probe was placed into the 0.01 M CaCl_2_ or the water, respectively. Inductively coupled plasma emission spectroscopy (ICP-OES; model: Perkin Elmer Optima 5300 DV) was used to measure the copper (Cu), iron (Fe), zinc (Zn), aluminum (Al), calcium (Ca), sodium (Na), magnesium (Mg), and P content of the soil. McDonald et al. (1998) method was used to measure the content of potassium, phosphorus, and sulfur. An Elementar analyzer (model: Vario Macro CNS, Elementar, Germany) was used to measure the total N and C content. A combination of 20 g soil with 80 ml K_2_SO_4_ 0.5 M was used to measure the dissolved organic C; the analysis used an OI Analytical Aurora 1030 Wet Oxidation TOC Analyzer (College Station, TX, USA). The same mixture (20 g soil with 80 ml K_2_SO_4_ 0.5 M) was used for measuring the nitrate and exchangeable ammonium; the content of the nitrate was measured using the hydrazine reduction method (Kempers and Luft, 1988), and the exchangeable ammonium was measured using the salicylate–nitroprusside method (Searle, 1984). The apparatus used was an automated flow injection Skalar auto analyzer, model: San plus (Skalar Analytical, Netherlands). All of the methods mentioned above were performed in triplicate, and the results are shown in **Table 1**.

**Table 1:** Chemical properties of soil collected from Murdoch University land field.

Chemical Content	Mean	SE (standard error)
Ammonium nitrogen mg/kg	3.00	0.00
Nitrate nitrogen mg/kg	20.00	4.00
Phosphorus Colwell mg/kg	19.50	2.50
Potassium Colwell mg/kg	24.50	1.50
Sulfur mg/kg	6.75	1.65
Organic carbon %	2.03	0.59
Conductivity dS/m	0.07	0.01
pH (CaCl_2_)	5.70	0.10
Copper mg/kg	0.50	0.06
Iron mg/kg	20.99	2.02
Manganese mg/kg	6.06	0.69
Zinc mg/kg	8.00	0.60
Aluminum meq/100 g	0.03	0.00
Calcium meq/100 g	8.17	0.60
Magnesium meq/100 g	0.48	0.10
Potassium meq/100 g	0.05	0.01
Sodium meq/100 g	0.06	0.02
Boron Hot CaCl_2_	0.27	0.04

### Experimental Design

A glasshouse experiment was conducted with a minimum temperature of 12°C during the night and a maximum temperature of 23°C during the day to investigate the effects of dried MA biomass and PPB on soil and the growth parameters of common pasture ryegrass (*Lolium rigidum* Gaudin). Treatments were defined as follows: 1) a dried mass of MA including *Chlorella* sp. and *Scenedesmus* sp., 2) a dried mass of PPB, 3) Black Marvel (a chemical fertilizer; see **Table S2**), 4) Hoagland fertilizer or Hoagland solution (Hoagland and Arnon, 1950), and 5) a negative control whereby no fertilizer was applied. A completely randomized design was used for eight replications (pots) of each treatment. Harvesting was performed at two different times, 45 and 60 days after emergence (DAE). We used two time periods to assess how the responses to organic fertilizers are influenced over time; as these fertilizers release nutrients *via* microbial mineralization, we aimed to assess this using phylogenetic investigation of communities by reconstruction of unobserved states (PICRUSt) and focusing on C and N putative gene count. During each harvest, four pots were harvested.

All treatments and chemical fertilizers were adjusted to deliver 100 kg N ha^−1^. Based on their compositions, different amounts of fertilizer were added to the soil, specifically, 2.2 g per pot of MA, 1.5 g per pot of PPB, and 1.1 g per pot of Black Marvel fertilizer. Before potting, these fertilizers were mixed with the soil using a potting soil mixer. The pots (with diameters of 13 cm at the top-rim and with plastic bags) were filled to 1.302 ± 0.001 kg. Before potting, seeds of common pasture ryegrass were soaked in deionized water for 3 days. Eight pre-germinated seeds of *Lolium rigidum* Gaudin were planted in each pot. The plants were thinned to four per pot after 10 DAE. Soil water capacity was maintained at 75% throughout this experiment. For this reason, the pots were weighed and watered every second day.

After each harvest, the height and the fresh weight of the shoots and roots were measured, and the plant material was oven-dried at 70°C for 72 h to ascertain the dry weight of the shoot and root samples. Bulk soil from the pots was analyzed for its chemical contents, and rhizosphere soil was collected for DNA extraction (see Section 2.4). The harvesting process was the same for both harvests.

### DNA Extraction From Rhizosphere Soil, PCR Amplification, and Sequencing

After removing the bulk soil, the rhizosphere soil was separated from the roots by gently shaking them. About 1 g of rhizosphere soil from each pot was collected in 1 ml microtubes and stored at −20°C until DNA extraction. DNA isolation was performed using the PowerSoil® DNA Isolation Kit (MoBio, Carlsbad, CA, USA) and by following the kit manual. Extracted DNA was quantified and adjusted (Qubit, Life Technologies, Australia) to 1 ng/μl using molecular-grade water and stored at −20°C until amplification. The amplification of the target 16S rRNA genes followed Mickan et al. (2018) using 27F and 519R bacterial primers (Caporaso et al., 2010; Mori et al., 2014) amended by the barcodes of Golay (Caporaso et al., 2012) with negative controls.

### Bioinformatics and PICRUSt

Paired-end reads were assembled by aligning the forward and reverse reads using PEAR (version 0.9.5) (Zhang et al., 2014). The primers were identified and trimmed. Trimmed sequences were processed using Quantitative Insights into Microbial Ecology (QIIME 1.8) (Caporaso et al., 2010) Usearch (version 8.0.1623; Edgar, 2010; Edgar et al., 2011) and UPARSE software. Using the Usearch tools, sequences were quality-filtered, and full-length duplicate sequences were removed and sorted according to abundance. Singletons or unique reads in the data set were discarded. Sequences were clustered according to a chimera that was filtered using the “rdp_gold” database as a reference. To obtain the number of reads in each operational taxonomic unit (OTU), the reads were mapped back to the OTUs with a minimum identity of 97%. QIIME taxonomy was assigned using the Greengenes database (version 13_8, Aug 2013; DeSantis et al., 2006). PICRUSt was used to predict a normalized gene count of key C and N gene encoding enzymes (see Mickan et al., 2018) using the above Greengenes database. PICRUSt uses evolutionary modeling to predict metagenomes from 16S data in relation with a reference genome database (Langille et al., 2013). The metagenomes were collapsed into the Kyoto Encyclopedia of Genes and Genomes (KEGG, http://www.kegg.jp/). 

### Statistical Analysis

Data analyses were conducted in the R-statistical environment (R Core Team, 2015), and a two-way analysis of variance (ANOVA) was used to test for the influence of fertilizer and harvesting on plant growth, soil chemistry, bacterial relative abundance, alpha diversity, and the putative functional genes associated with C and N cycling. *Post-hoc* analysis was performed using a Tukey HSD test on the fertilizer during only those harvests where the two-way ANOVA analysis yielded significant results. When the response variables displayed homogeneous errors in the variance of residuals, a logarithmic or square root transformation was performed. The observed richness was calculated based on the number of OTUs detected in each sample, and the coverage was calculated using Good (1953) coverage estimator. A permutational multivariate analysis of variance (PERMANOVA) was used to test the significant differences between taxonomic bacteria levels (OUT levels) and treatments (fertilizer and harvesting) in the vegan package (Oksanen et al., 2013) based on the Bray–Curtis distances calculated from the relative abundances of OTUs at 97% similarity.

## Results

### Growth Parameters

The analysis of plant growth parameters at the first harvest (45 DAE) revealed that the PPB, MA, and Black Marvel fertilizers had a positive effect on root length, while the addition of Black Marvel resulted in significantly longer roots than in the control plants (29%, *P* = 0.011) (**Table 2**). The shoot fresh weight in the plants treated with PPB (54%, *P* = 0.002), Hoagland (41%, *P* = 0.017), and Black Marvel (78%, *P* < 0.001) was significantly higher than in the control plants (**Table 2**). Further, the shoot dry weight showed significant enhancement under the Black Marvel (77%, *P* < 0.001), PPB (47%, *P* = 0.019), and Hoagland (44%, *P* = 0.033) fertilizer treatments (**Table 2**).

**Table 2:** The effect of different fertilizers on growth parameters of *Lolium rigidum* Gaudin after first and second harvests (mean ± SE, n = 4). Different letters show significant differences (Tukey HSD, *P* < 0.05).

	Harvest	MA	PPB	B_Marvel	Hoagland F	Control
Shoot height (cm)	1	36.4 ± 2.2 a	40.6 ± 2.6 c	47.2 ± 1.0 b	40.0 ± 2.3c	40.2 ± 0.7 c
Root height (cm)	1	33.5 ± 2.3 c	31.6 ± 0.6 c	37.7 ± 0.9 b	27.2 ± 2.0 a	29.1 ± 1.4 a
Shoot fresh weight (g)	1	12.2 ± 0.5 a, b	14.3 ± 0.4 b, c	16.6 ± 1.3 c	13.2 ± 0.7 b	9.3 ± 0.3 a
Root fresh weight (g)	1	13.9 ± 1.1 a	11.0 ± 1.0 a	11.7 ± 0.5 a	9.1 ± 1.1 a	11.5 ± 1.6 a
Shoot dry weight (g)	1	1.6 ± 0.1 a, b	1.9 ± 0.1 b, c	2.2 ± 0.2 c	1.8 ± 0.2 b, c	1.3 ± 0.1 a
Root dry weight (g)	1	2.1 ± 0.1 a	2.0 ± 0.2 a	2.3 ± 0.2 a	1.6 ± 0.2 a	2.0 ± 0.4 a
Shoot height (cm)	2	38.8 ± 0.5 a	40.2 ± 1.1a	37.8 ± 2.4a	39.3 ± 3.1a	35.4 ± 2.7 a
Root height (cm)	2	43.3 ± 1.6 a	39.0 ± 1.4 a	35.8 ± 1.8 a	36.5 ± 2.4 a	38.3 ± 1.1 a
Shoot fresh weight (g)	2	17.0 ± 1.1 a, b	21.3 ± 1.2 b, c	23.3 ± 2.8 b, c	27.6 ± 0.6 c	12.6 ± 1.0 a
Root fresh weight (g)	2	33.6 ± 5.4 a	28.6 ± 3.2 a	25.5 ± 2.2 a	28.0 ± 4.9 a	22.1 ± 2.6 a
Shoot dry weight (g)	2	3.4 ± 0.3 a, b	4.3 ± 0.3 b, c	5.2 ± 0.5 c	5.6 ± 0.2 c	2.3 ± 0.1 a
Root dry weight (g)	2	6.4 ± 1.9 a	5.0 ± 1.8 a	4.2 ± 0.5 a	4.2 ± 1.5 a	4.6 ± 1.7 a

MA, microalgae; PPB, purple phototrophic bacteria; B_Marvel, Black Marvel.

The analysis of growth data from the second harvest showed that the application of organic (PPB and MA) and chemical fertilizers (Hoagland and Black Marvel) positively affected the shoot fresh and dry weights (**Table 2**). In contrast to the control, PPB, Black Marvel, and Hoagland increased plant shoot fresh weight by 68% (*P* = 0.008), 84% (*P* = 0.001), and 118% (*P* < 0.001), respectively. Additionally, following 60 days of treatment, the PPB (84%, *P* = 0.003), Black Marvel (122%, *P* < 0.001), and Hoagland (139%, *P* < 0.001) fertilizers had significantly positive effects on the shoot dry weight of the plants. Nevertheless, there were no significant changes for other growth parameters, such as shoot and root height, root fresh weight, and root dry weight under the different fertilizer treatments (**Table 2**).

### Soil Properties

The macronutrients in the bulk soil samples after the first harvest showed a significant increase in N in soils treated with MA (*P* = 0.016) (**Supplementary Table S3**). Further, the amounts of phosphorus (P) in the soils of MA (37%, *P* = 0.031), PPB (54%, *P* = 0.002), and Hoagland (45%, *P* = 0.009) and the amount of Mg in soils treated with PPB (*P* = 0.019) also increased significantly, suggesting a solubilization of organic bound P from the biomass (**Supplementary Table S3**). In terms of micronutrients, the soil of pots treated with organic fertilizers revealed higher contents of Fe, Cu, and manganese than the control and chemical fertilizers. The MA, PPB, and Hoagland treatments, respectively, had 153% (*P* < 0.001), 159% (*P* < 0.001), and 100% (*P* = 0.002) more Fe than the control (**Supplementary Table S3**). The Cu and Mn contents of the soil treated with the MA, PPB, and Hoagland fertilizers were also increased (**Supplementary Table S3**).

Bulk soil analysis of pots during the second harvest revealed a significant increase in the K content of the pots treated with MA (69%, *P* = 0.001), PPB (100%, *P* < 0.001), and Hoagland fertilizer (84%, *P* < 0.001) in contrast to the control (**Supplementary Table S4**). Unlike the first harvest, the results of the soil analysis for the second harvest showed that the amount of Fe in the Hoagland-treated soil (−45%, *P* = 0.047) and the level of Cu in all the treatments, including MA (−53%, *P* = 0.002), PPB (−58%, *P* = 0.001), Black Marvel (−42%, *P* = 0.011), and Hoagland (−60%, *P* = 0.001), was significantly lower than in the control (**Supplementary Table S4**).

### Rhizosphere Bacterial Community Assemblage

#### Alpha Diversity

A two-way ANOVA revealed significant effects of fertilizer on different diversity indices, including inverse Simpson (*P* = 0.030), Fisher (*P* = 0.005), richness (*P* = 0.020) and evenness (*P* = 0.001) (**Supplementary Table S** **5**). There was a significant increase in alpha bacterial diversity in the rhizosphere soil of plants treated with MA measured by the Fisher (25%, *P* = 0.018) and richness (73%, *P* = 0.001) indices (**Table 3**) during the second harvest. Further, the richness of the rhizosphere bacteria under the PPB (55%, *P* = 0.030) and Black Marvel (52%, *P* = 0.045) treatments was significantly higher than that of the control (**Table 3**).

**Table 3:** Diversity indices of soil bacteria in different fertilizer treatments (MA, PPB, Hoagland, Black Marvel, and without fertilizer). Harvest 1 was 45 days after emerging plants, and harvest 2 was 60 days after emerging the plants. Values are the mean for each treatment and SE of the mean (n = 4).

		Harvest 1					Harvest 2				
		Control	MA	PPB	Hoagland	B_Marvel	Control	MA	PPB	Hoagland	B_Marvel
Fisher	Mean	637.579	637.937	602.884	622.257	490.912	579.069	727.854	708.462	615.424	661.136
	SE	30.156	38.648	35.777	30.316	19.684	8.495	21.076	12.815	25.158	34.182
Richness	Mean	1,832.000	1,792.750	1,786.750	2,052.500	1,333.250	1,424.250	2,467.250	2,210.750	1,871.500	2,174.000
	SE	206.567	189.965	148.684	194.103	149.006	108.098	104.706	73.253	134.842	169.518
Evenness	Mean	0.854	0.848	0.837	0.826	0.837	0.860	0.843	0.834	0.835	0.832
	SE	0.003	0.001	0.007	0.003	0.011	0.0010	0.005	0.004	0.004	0.004
Inverse Simpson	Mean	194.744	160.614	151.481	155.040	108.031	163.446	206.140	171.102	154.005	166.242
	SE	14.739	16.815	24.997	13.889	5.670	9.263	6.892	10.556	14.813	21.782

#### Relative Abundance

**Figure 1:**
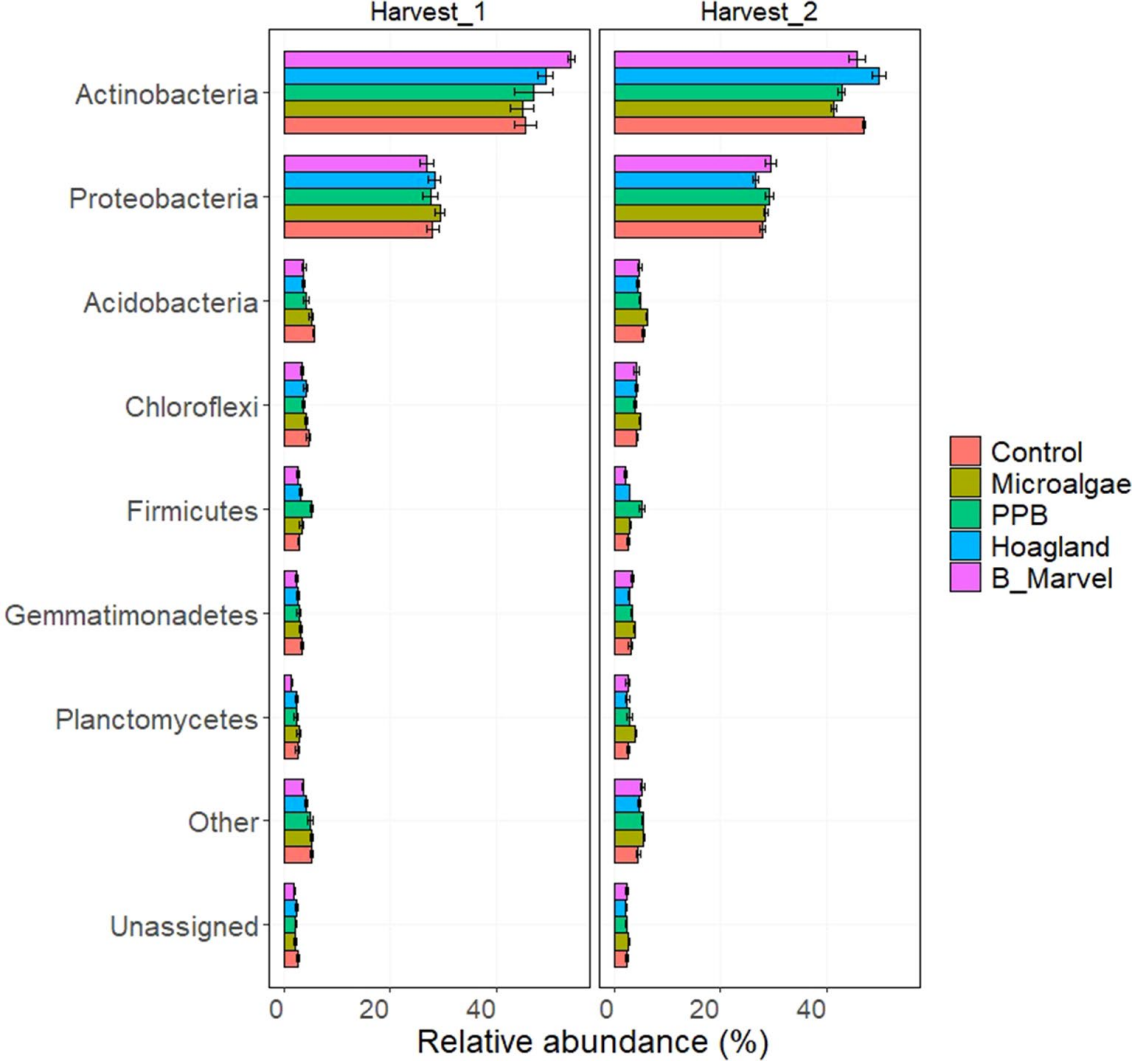
Relative abundance of bacteria phyla in rhizosphere soil of control, microalgae, purple phototrophic bacteria, Hoagland, and Black Marvel, after 45 (first harvest) and 60 (second harvest) days from emerging the plants.

The relative abundance at the phylum level of rhizosphere bacteria during the first harvest was dominated by the phyla Actinobacteria and Proteobacteria in all of the control and treatment soils (**Figure 1**). The relative abundance of Firmicutes in the PPB treatment was significantly higher than in the control at the first (87%, *P* < 0.001) and second harvests (94%, *P* < 0.001) (**Supplementary Table S6**, **Figure 1**). During the second harvest, the relative abundance of Actinobacteria was significantly lower in the MA treatment than in the Hoagland treatment (−17%, *P* = 0.041). In contrast, the relative abundance of Acidobacteria (40%, *P* = 0.015) and Planctomycetes (66%, *P* = 0.020) in the rhizosphere soil of pots treated with MA was significantly higher than it was in Hoagland fertilizer (**Supplementary Table S6**, **Figure 1**).

#### The Community-Level Effect of Different Fertilizers on Bacteria OTUs

**Figure 2:**
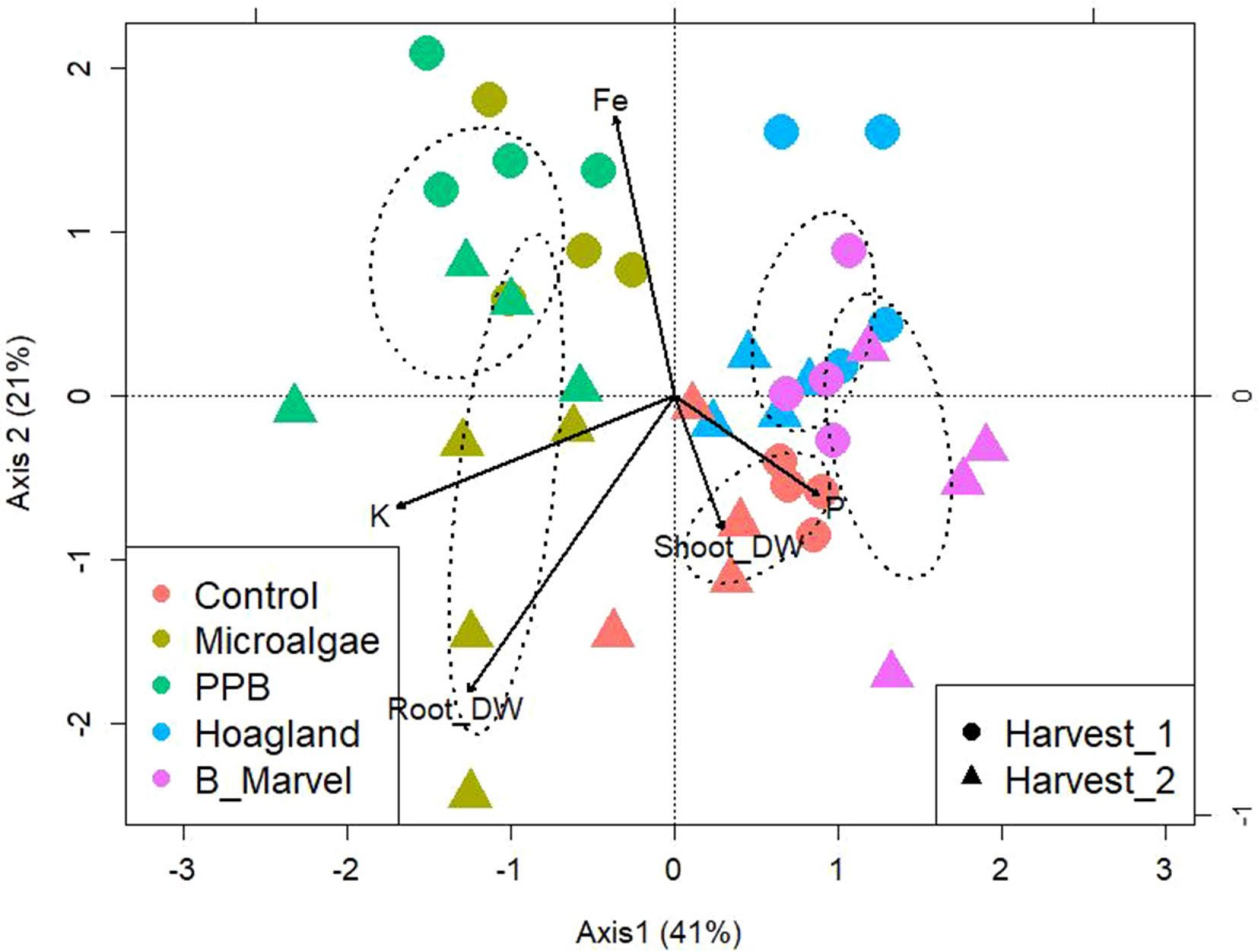
Canonical correspondence analysis of bacteria OTUs from rhizosphere soil of control, microalgae, PPB, Hoagland, and Black Marvel treatments, with soil chemical characters and plant growth parameters during first and second harvests.

To evaluate the influence of the organic fertilizer on the community OTU-level distribution, a PERMANOVA was performed. Analysis by PERMANOVA at a 97% OTU-level similarity reveals the significant effects of fertilizer (*P* = 0.013) and harvest (*P* = 0.043) and the interaction between these factors: fertilizers*harvest (*P* = 0.003) (**Supplementary Table S7**). To establish what was driving the interaction between soil bacteria and environmental variables, a canonical correspondence analysis (CCA) of OTUs with soil chemical characters and plant growth parameters was performed (**Figure 2**). The data were clustered into four groups. The microbial composition of the PPB and MA from the first harvest was clustered together as a result of increasing soil Fe content, while the microbial community from the second harvest of MA was categorized alone according to the potassium (K) content of the soil and the root dry weight (**Figure 2**). The microbial communities in the control samples were separated from the other groups based on the soil’s P content and shoot dry weight (**Figure 2**). Finally, the two chemical fertilizers were clustered together (**Figure 2**).

### Rhizosphere Bacterial C and N Pathway Genes

The results of the ANOVA during the first harvest revealed a significant effect of fertilizer on the N-cycling genes *hao* (*P* = 0.016), *narG* (*P* = 0.019), *nrfA* (*P* = 0.001), *nirK* (*P* = 0.022), and *nosZ* (*P* = 0.010) (**Supplementary Table S8**). Harvest time and fertilizer treatment also influenced the C-degrading genes alpha-amylase (*P* = 0.007), glucoamylase (*P* = 0.047), beta-glucosidase (*P* = 0.048), chitinase (*P* = 0.044), and catalase (*P* = 0.035) (**Supplementary Table S9**).

**Figure 3:**
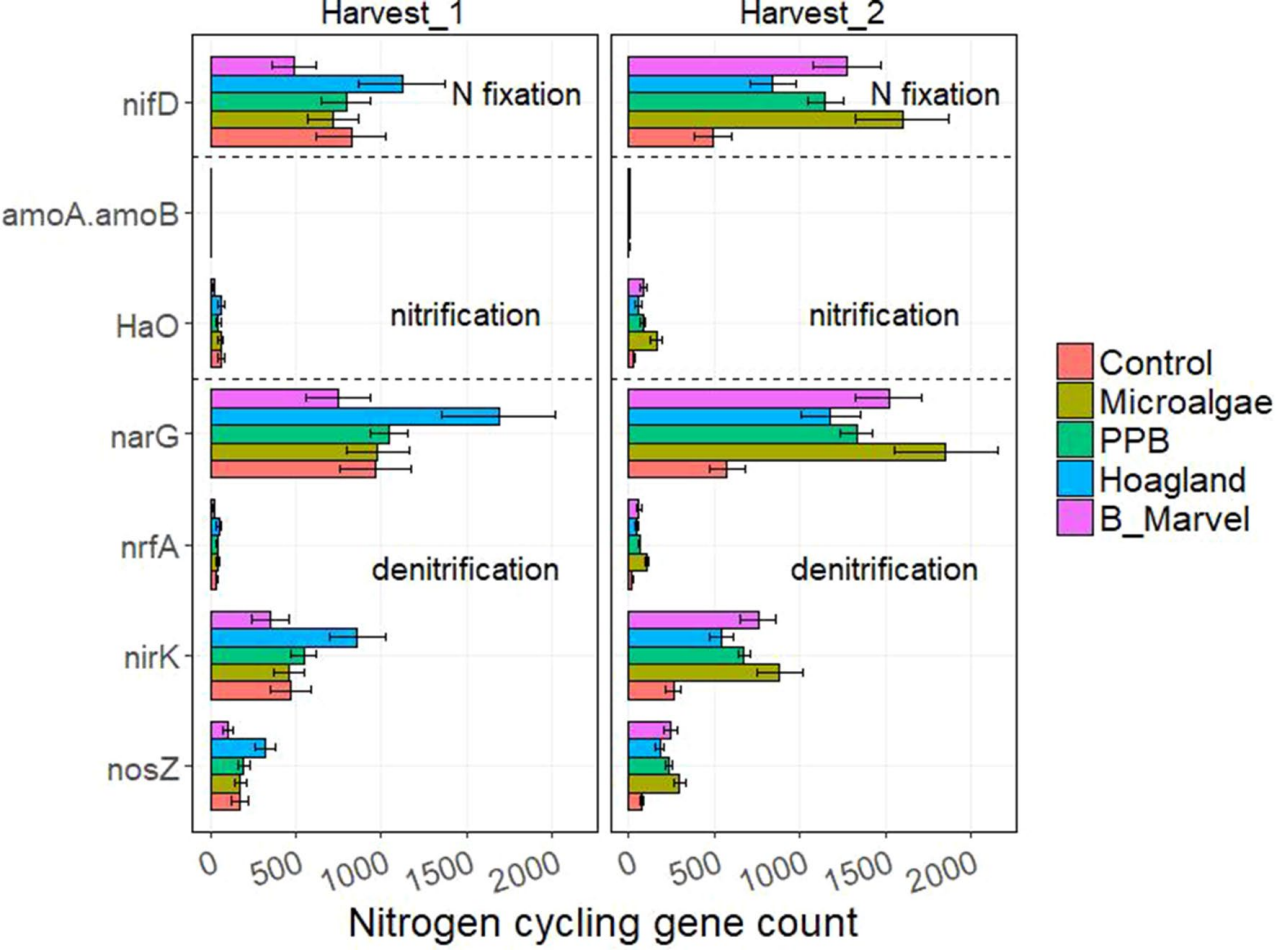
Scale of nitrogen-cycling genes from control, microalgae, PPB, Black Marvel, and Hoagland fertilizer during first and second harvests.

**Figure 4:**
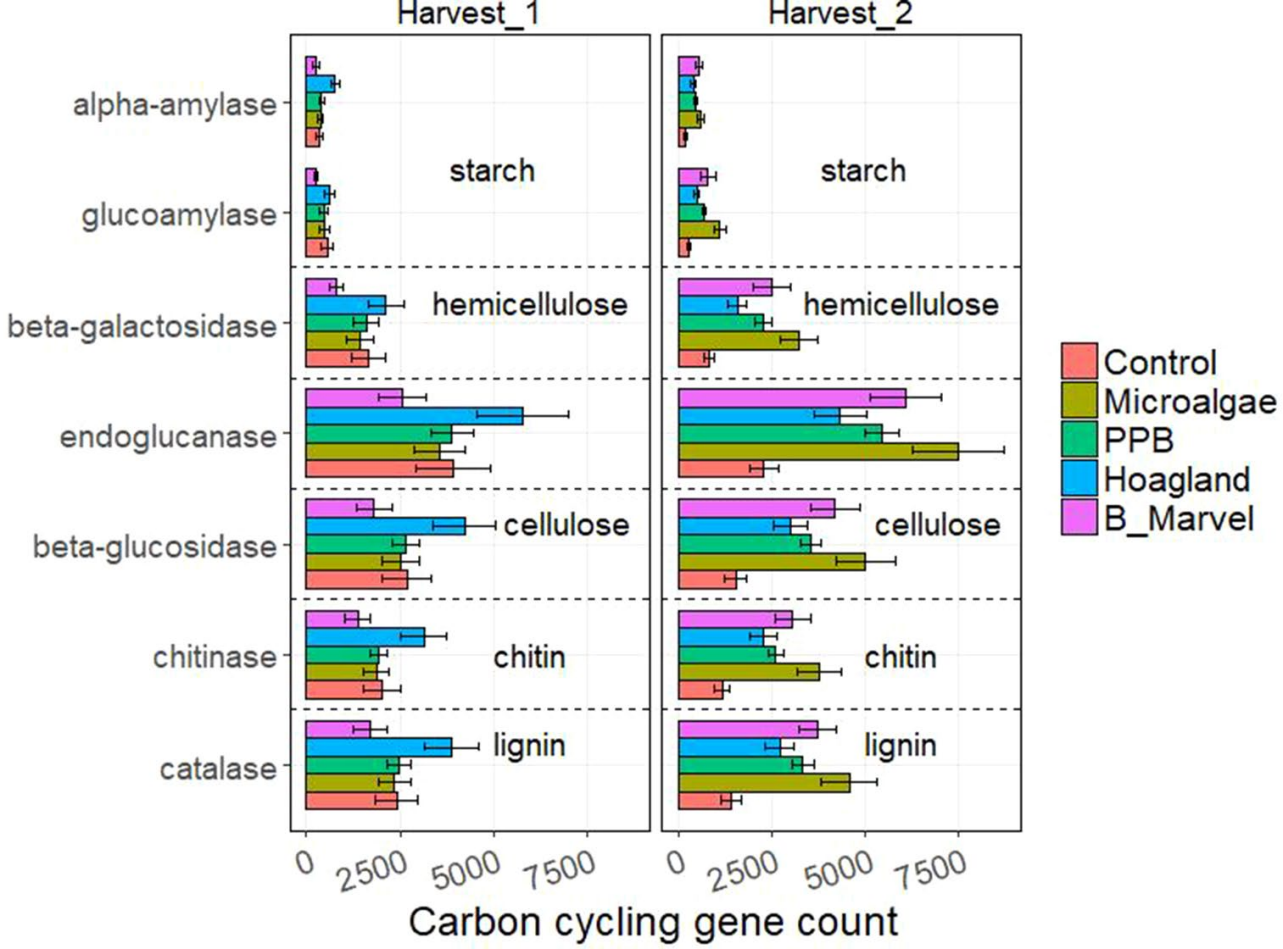
Scale of carbon-cycling genes from control, microalgae, PPB, Black Marvel, and Hoagland fertilizer during first and second harvest.

Tukey *t* test results for the N- and C-cycling genes showed that Hoagland fertilizer was associated with a significant increase in alpha-amylase genes during the first harvest (115%, *P* = 0.022) (**Figures 3** and **4**).

During the second harvest, the abundance of *nifD*, the N-fixing gene, in the MA treatment was greater than in the other treatments and the control (227%, *P* = 0.004) (**Figure 3**). Further, the abundance of the nitrification gene *hao* (453%, *P* = 0.001) and the denitrification genes *narG* (223%), *nrfA* (433%, *P* < 0.001), *nirK* (239%, *P* = 0.006), and *nosZ* (286%, *P* = 0.006) were significantly greater in the MA treatment than in the control and the other treatments (**Figure 3**).

All of the measured C-degrading genes, including alpha-amylase (198%, *P* = 0.027), glucoamylase (291%, *P* = 0.001), beta-galactosidase (288%, *P* = 0.002), endoglucanase (226%, *P* = 0.004), beta-glucosidase (222%, *P* = 0.004), chitinase (218%, *P* = 0.003), and catalase (221%, *P* = 0.003), were more abundant in the MA treatment (**Figure 4**).

## Discussion

### Plant Growth Parameters

This experiment evaluated the effects of the novel organic fertilizers PPB and MA as a means of recovering nutrients from waste resources and comparing them with two synthetic fertilizers (Hoagland and Black Marvel) as positive controls. Overall, the application of MA and PPB did not increase the shoot dry biomass relative to the two positive controls after 60 days. Nevertheless, shoot dry biomass was only slightly lower in the plants receiving the PPB biomass than in those treated with the Hoagland solution (a 20% reduction), which had the greatest yield (see **Table 2**). This indicates that PPB is an appropriate source of macro- and micronutrients (up to 80%) for common pasture ryegrass and could function as an alternative to chemical fertilizers in agro-ecosystems. To date, there is little published on the effects of dried PPB on plant growth; however, other researchers have reported the positive effects of fresh PPB on plant weight and grain yield (Lee et al., 2008; Harada et al., 2005). Enhanced crop performance has been attributed to the presence of phytohormones (e.g., auxins) in PPB, the promotion of microbial activity (particularly, N fixation), and an increase in soil dehydrogenases (Lee et al., 2008; Wu et al., 2013; Wong et al., 2014). In this study, PPB did not affect the abundance of the N- and C-cycling genes relative to the positive controls (**Figures 3** and **4**); however, there was a marked increase in the abundance of Firmicutes under PPB (**Figure 1**). Representatives of the phyla Firmicutes have been shown to form endophytic associations with plants that promote growth and health (Xia et al., 2015); perhaps this is one of the mechanisms by which PPB stimulated the common pasture ryegrass.

In contrast, MA (*Chlorella* and *Scenedesmus*) did not perform as well as the PPB, which was surprising, as other studies have reported MA to be comparable to a chemical fertilizer (Mulbry et al., 2005; Schreiber et al., 2018). One possible explanation is that the treatments in the present study were adjusted to the same N rate (100 kg N ha^−1^), whereas in the other studies, the treatments were fixed on P (Schreiber et al., 2018), indicating that the MA treatment was P limited. Plants tend to grow more root biomass under P-limiting conditions to increase the rate of P uptake (Schachtman et al., 1998; Wang et al., 2016). Perhaps this explains why the most significant root biomass and root weight in both harvests belonged to the MA treatment (**Table 2**). This accords with the results of Shaaban (2001), who showed that using MA as a bio-fertilizer enhanced plant productivity by increasing the root volume to maximize mineral uptake and nutrient absorption. In this investigation, we mostly focused on the effects of PPB and MA dried matter on the soil’s bacterial community and especially on nutrient cycling.

### The Rhizosphere Bacterial Community

Another major focus of this study was to investigate the effects of PPB and MA dried matter on the soil’s bacterial community and nutrient cycling. Our study revealed a significant increase in bacterial OTU richness in the rhizospheric soil treated with MA and PPB organic fertilizers relative to the control (**Table 3**). Increased bacterial diversity (as indicated by various diversity indices) in response to organic soil amendment has been widely reported before, and it has been shown that bacterial communities in organically managed soils are typically more diverse than conventional inorganic fertilized agro-ecosystems (Sun et al., 2004; Peacock et al., 2001; He et al., 2008; Zhong et al., 2010; Bonilla et al., 2012; Herzog et al., 2015). Changes in the soil’s microbial diversity are related to the increased availability of both organic C and a mineral input (such as N and P) supplied in the organic amendments (Zhong et al., 2007; Fierer et al., 2012). When labile C enters the soil (via organic amendments), a substrate-induced succession of microorganisms from r- to k-strategist (Fontaine et al., 2003; Blagodatskaya et al., 2007) occurs. Initially, fast-growing r-strategists (copiotrophs), which can maximize their growth rate on labile substrates, dominate following organic amendments (Fontaine et al., 2003; Jenkins et al., 2010). This explains why the addition of MA and PPB to soil leads to increased community richness but also to decreased evenness (**Table 3**), and this has been observed previously following long-term (Hartmann et al., 2015) and short-term (Fierer et al., 2012) applications of organic matter. Eventually, once the labile C substrates have been exhausted, the copiotrophs are superseded by the slow-growing k-strategists (oligotrophs). Oligotrophs utilize resources more efficiently by degrading recalcitrant SOM (Fontaine et al., 2003; Fierer et al., 2012) and prefer nutrient-poor environments (Ren et al., 2016).

Comparisons between the different treatments showed a significant increase in the relative abundance of Firmicutes in PPB-amended soil for both harvests (**Supplementary Table S6**, **Figure 1**). As copiotrophs (r-strategists), Firmicutes have evolved survival strategies such as high growth rates and metabolic versatility to compete for C resources, particularly labile C (Fierer et al., 2007). They are capable of degrading a variety of simple mineralizable and complex organic materials (Hartmann et al., 2015; Whitman et al., 2016) and therefore thrive in nutrient-rich environments where they out-compete other slow-growing phyla (Fierer et al., 2007). Since their population is enhanced by the availability of a C source (Eilers et al., 2010; Fierer et al., 2012), PPB is likely an appropriate source of labile C. Firmicutes are often isolated from soils receiving C-rich organic amendments, such as manure or compost (Poulsen et al., 2013; Francioli et al., 2016).

Aside from Firmicutes, there were limited changes in the relative abundance of other bacterial phyla across the different treatments and the control samples, and this could reflect the short-term duration of this experiment. However, there was a slight increase in gram-negative bacteria associated with the organic amendments during the second harvest as opposed to the first harvest (**Figure 1**), including Proteobacteria (MA and PPB), Acidobacteria (MA), Chloroflexi (MA) and Gemmatimonadetes (MA). The occurrence of gram-negative bacteria in rhizosphere soil is more common than that of gram-positive bacteria (Elo et al., 2000; Söderberg et al., 2004). Moreover, a change in the prevailing soil conditions from oligotrophic to copiotrophic is usually accompanied by an increase in the relative abundance of gram-negative bacteria in the soil (Borga et al., 1994; Saetre and Bååth, 2000). For example, there was a shift from gram-positive to gram-negative bacteria following the addition of compost (Mickan et al., 2018). A number of gram-negative bacteria exhibit r-strategy traits and are therefore more abundant in nutrient-rich environments (deVries and Shade, 2013).

Addition of organic matter to soil also influences plant growth indirectly, by changing the soil’s bacterial communities, enzyme activity, and C cycling (Sonia et al., 2011; Fernandez et al., 2016). There was a marked increase in the relative abundance of Actinobacteria in the rhizosphere of chemical fertilizer treatments and soil without any treatment (**Figure 1**). A dominance of Actinobacteria has been reported in soils receiving inorganic fertilizer and unamended soil (Dai et al., 2018; Mickan et al., 2018). Actinobacteria are known for their ability to degrade complex compounds and recalcitrant materials, such as starch, cellulose, and lignins (Ulrich et al., 2008; Jenkins et al., 2009). This might explain why there was a marked increase in the relative abundance of C-degrading genes, such as alpha-amylase, glucoamylase, beta-galactosidase, endoglucanase, beta-glucosidase, chitinase, and catalase in the chemical fertilizer treatment during the first harvest (**Figure 4**). Products of these genes are involved in decomposing the C products of organic matter, such as starch, hemicellulose, cellulose, chitin, and lignin. However, other studies have shown a higher relative abundance of Actinobacteria and other cellulolytic bacteria in organic amended soil, such as compost (Toyota and Kuninaga, 2006; Ulrich et al., 2008; Zhong et al., 2010); therefore, we hypothesized that there would be an increase in Actinobacteria and associated C genes in the organic fertilizer treatments. During the second harvest (**Figure 4**), the predicted abundance of C-degrading genes was highest in the MA treatment and lowest in the PPB treatment. This suggests that there is a greater abundance of readily degradable, labile forms of C in PPB amendments (Jenkins et al., 2010). In contrast, the MA samples probably contain more recalcitrant forms of C, similar to those found in composts (Jenkins et al., 2010; Zhong et al., 2010).

### N-Cycling Pathways

Another possibility to account for the unexpectedly low yield obtained from the MA treatment is the fact that dried MA biomass has been reported to act as a slow-release fertilizer (Mulbry et al., 2005; Coppens et al., 2016; Wuang et al., 2016). The most N content of MA is in the form of organic N; therefore, only 3% of the total N will be available to the plant at the time of application (Mulbry et al., 2005). This means that the organic N fraction in MA must first be mineralized to ammonium N (Pan et al., 2014) using microorganisms that rely on the N for their growth (Parfitt et al., 2005). Our study showed a marked increase in the predicted abundance of putative genes involved in N-cycling pathways, including N fixation (*nifD*), nitrification (*hao*), and denitrification (*narG*, *nirK*) in the MA-treated soil during the second harvest (**Figure 3**). However, only *hao* and *nrfA* were significantly more abundant following the MA treatment as opposed to other treatments. Hydroxylamine oxidoreductase (*hao*), together with ammonia monooxygenase (*Amo*), catalyzes the first step of nitrification (Ren et al., 2018) through which ammonia-oxidizing bacteria oxidize ammonia into nitrite (Hallin et al., 2009). Ammonia-oxidizing bacteria and archaea are mainly affiliated with Proteobacteria (Wessén et al., 2010); accordingly, there was a slight increase in the abundance of both these bacteria in the MA samples from the first harvest. A nitrification pathway comprises two steps—ammonia oxidation and nitrite oxidation (Ren et al., 2018)—and the second step converts nitrite into nitrate, the preferred form of N for plant uptake (Maathuis, 2009).

In contrast, nitrite reductase (NrfA) does the reverse by catalyzing nitrite to ammonium during the process of dissimilatory nitrate reduction to ammonium, or DNRA (Welsh et al., 2014). This implies that there is a higher potential for nitrification as well as N retention (DNRA) in soils receiving MA. Thus, by removing the nitrite from the soil before it has a chance to be oxidized to nitrate, the DNRA bacteria are in direct competition with the plant for mineral N. Increased N retention in the soil (via DNRA) could, therefore, affect plant growth by reducing the availability of nitrate. However, plants can also utilize ammonium (Maathuis, 2009), and the immobilization of ammonium by the plants’ N efflux, DNRA, and microbial biomass N (MBN) turnover was shown to be equal to at least 35% of the nitrification rate (Burger and Jackson, 2005). Thus, N retention in soils may actually enable plants to capture more ammonium than was previously thought (Burger and Jackson, 2005).

The application of PPB to the soil had less effect on the N-cycling genes than that of MA. Nevertheless, one of the N-cycling genes enhanced in both the PPB- and MA-treated soil and not in the Hoagland-treated soil was *nifD* (a subunit of nitrogenase molybdenum–iron protein). Nitrogenase catalyzes the conversion of atmospheric N to ammonium in a process called N fixation. A number of N-fixing taxa are affiliated with Proteobacteria and Firmicutes (Ren et al., 2018), and, in our experiment, these bacteria were more abundant in both organic fertilizer treatments (**Figure 1**).

Denitrification is a key process of N cycling during which nitrate is converted to nitric oxide, nitrous oxide, and di-nitrogen (Ren et al., 2018). Previous studies have shown higher rates of denitrification in organic, amended soil than in inorganic, amended soil (Enwall et al., 2005). Although, there was a trend toward an increased abundance of the denitrification genes *narG*, *nirK*, and *nosZ* in the MA-amended soil, this was not significantly different from the other treatments, especially that of the Black Marvel (**Figure 3**). However, Acidobacteria, Proteobacteria, and Firmicutes were more abundant in the MA- and PPB-amended soil than in the soil that received the other treatments and the control during the second harvest (**Figure 3**); further, denitrifying genes have been found in bacterial strains affiliated with these phyla (Lladó et al., 2017).

## Conclusions

Two novel organic fertilizers were evaluated as potential bio-fertilizers; one, PPB, almost performed as well as the inorganic fertilizers, implying that it could meet the nutritional requirements of crops. Enhanced crop performance was attributed to changes in diversity and an abundance of microorganisms resulting from an increased availability of labile C. In particular, there was a marked increase in the abundance of copiotrophic Firmicutes associated with the PPB treatment. Some members of this phylum are able to act as biocontrol agents against plant pathogens, promoting plant growth and health.

Although MA did not perform as well as PPB, this is plausibly a consequence of the high N content of MA in the form of organic N, which would degrade slowly in soil, resulting in slower, more controlled release of nutrients. This makes MA potentially interesting as a slow-release fertilizer for horticulture, pasture, and the rehabilitation of disturbed land. The application of MA to soil also had a profound effect on C and N cycling. One feature of this dynamic was the increased potential for N mineralization and nitrification, coupled with N retention (DNRA). Management practices that enhance C sequestration and N retention in agricultural soils, while limiting N losses from nitrification *via* greenhouse gas emissions and leaching, should be encouraged. Future studies are recommended to test MA as a slow-release fertilizer amendment.

Overall, MA and PPB grown on agri-industrial wastewaters could be developed as effective organic fertilizers. Recovery and reuse of these products on pasture and cropping enterprises has the potential to increase on-farm sustainability, productivity, and profitability.

## Data Availability Statement

All datasets generated for this study are included in the manuscript/**Supplementary Files**.

## Author Contributions

BM and NM supervised the work. BM analyzed the data and helped in bioinformatics and performing the experiment. SZ performed the experiment and measurements and wrote the manuscript. BM and TH provided the dry matter of microalgae and purple phototrophic bacteria. BM, SJ, and TH contributed to the writing. BM, SJ, TH, HR, and NM revised the paper. All authors read and approved the manuscript and agreed with authorship and submission of the manuscript.

## Funding

This research was funded by Richgro garden products (http://richgro.com.au/) with financial support by the Department of Industry and Science: Innovations Connections Grant: ICG000114. The salaries of SJ were partially supported by “RnD4Profit-14-1-022—Waste to Revenue: Novel Fertilisers and Feeds,” Australian Pork Limited and the Australian government (Department of Agriculture and Water Resources), as part of the Rural Research and Development (R&D) for Profit program.

## Conflict of Interest

The authors declare that the research was conducted in the absence of any commercial or financial relationships that could be construed as a potential conflict of interest.
