# Supplementary material for: Microalgae and Phototrophic Purple Bacteria for Nutrient Recovery From Agri-Industrial Effluents: Influences on Plant Growth, Rhizosphere Bacteria, and Putative Carbon- and Nitrogen-Cycling Genes
Source: Front Plant Sci. 2019 Sep 27;10:1193. doi: 10.3389/fpls.2019.01193 (PMC6779020; doi:10.3389/fpls.2019.01193)
Supplement: Supplementary file 1 [file DataSheet_1.docx]

Supplementary Material

# Supplementary Tables

**Supplementary Table S1.** Chemical properties of dried purple phototrophic bacteria (PPB) cultured on piggery effluent and microalgae (MA) cultivated on food waste digestate

|  |  | Fe (mg/kg) | Cu (mg/kg) | Zn (mg/kg) | Mn (mg/kg) | K (Wt%) | Na (Wt%) | Mg (Wt%) | Ca (Wt%) | P (Wt%) | S (Wt%) | C (%) | H (%) | N (%) |
| --- | --- | --- | --- | --- | --- | --- | --- | --- | --- | --- | --- | --- | --- | --- |
| PPB | Mean | 1241.428 | 885.671 | 732.309 | 567.856 | 0.549 | 0.153 | 1.308 | 1.989 | 2.942 | 0.489 | 42.835 | 6.545 | 8.860 |
|  | SE | 10.560 | 23.005 | 20.360 | 4.296 | 0.001 | 0.002 | 0.013 | 0.016 | 0.010 | 0.006 | 0.705 | 0.085 | 0.240 |
| MA | Mean | 12634.837 | 86.351 | 1459.479 | 148.581 | 0.646 | 0.190 | 0.326 | 2.207 | 1.633 | 0.319 | 38.295 | 5.520 | 6.045 |
|  | SE | 30.372 | 1.193 | 5.550 | 2.546 | 0.007 | 0.008 | 0.003 | 0.028 | 0.003 | 0.002 | 1.775 | 0.270 | 0.295 |

**Supplementary Table S2.** Chemical component of Black Marvel chemical fertiliser.

| Chemical componet | W/W% |
| --- | --- |
| Nitrogen as ammonium | 11.7% |
| Nitrogen as nitrate | 0.85% |
| Total nitrogen | 12.55% |
| Phosphorus as water soluble | 0.82% |
| Phosphorus as citrate soluble | 0.20% |
| Total phosphorus | 1.02% |
| Potassium as sulphate | 6.6% |
| Potassium as chloride | 4.4% |
| Total potassium | 11.0% |
| Sulphur as sulphate | 16.1% |
| Calcium as sulphate | 2.2% |
| Copper as sulphate | 0.07% |
| Zinc as sulphate | 0.07% |
| Iron as sulphate | 2.0% |

**Supplementary Table S3.** Soil properties after 45 days treatment with different fertilisers.

|  | **MA** | **PPBacteria** | **Hoagland F** | **B_Marvel** | **Control** |
| --- | --- | --- | --- | --- | --- |
| C (%) | 2.990±0.292 | 3.063±0.252 | 3.375±0.374 | 2.593±0.680 | 2.825±0.381 |
| H (%) | 0.290±0.037 | 0.228±0.031 | 0.285±0.018 | 0.208±0.064 | 0.183±0.028 |
| N (%) | 0.200±0.062* | 0.128±0.055 | 0.090±0.018 | 0.040±0.031 | 0.000±0.000 |
| Fe (mg/kg) | 1598.465±116.593** | 1640.343±76.182** | 1265.683±147.343* | 803.498±20.606 | 631.635±23.138 |
| Cu (mg/kg) | 56.085±3.948** | 58.530±2.608** | 45.280±5.291* | 28.473±2.261 | 25.523±0.863 |
| Zn (mg/kg) | 27.530±6.315 | 31.518±2.821 | 33.175±8.401 | 17.785±1.098 | 16.213±1.304 |
| Mn (mg/kg) | 55.088±3.184** | 58.203±1.916** | 53.603±4.118** | 35.640±1.274 | 30.635±1.196 |
| K (wt%) | 0.004±0.001 | 0.005±0.000 | 0.002±0.001 | 0.003±0.003 | 0.000±0.000 |
| Na (wt%) | 0.023±0.002 | 0.025±0.001 | 0.022±0.001 | 0.023±0.003 | 0.020±0.000 |
| Mg (wt%) | 0.001±0.001 | 0.003±0.001* | 0.002±0.001 | 0.000±0.000 | 0.000±0.000 |
| Ca (wt%) | 0.182±0.015 | 0.222±0.018 | 0.238±0.006 | 0.173±0.029 | 0.170±0.010 |
| P (wt%) | 0.028±0.001* | 0.031±0.001* | 0.029±0.001* | 0.023±0.003 | 0.020±0.001 |
| S (wt%) | 0.000±0.000 | 0.003±0.003 | 0.018±0.009 | 0.035±0.005 | 0.028±0.004 |

* Significant at < 0.05 level, ** Significant at < 0.01 level

**Supplementary Table S4.** Soil properties after 60 days treatment with different fertilisers.

|  | **MA** | **PPBacteria** | **Hoagland F** | **B_Marvel** | **Control** |
| --- | --- | --- | --- | --- | --- |
| C (%) | 3.918±1.387 | 3.635±0.503 | 2.655±0.491 | 2.405±0.654 | 3.473±0.340 |
| H (%) | 0.280±0.127 | 0.230±0.060 | 0.180±0.065 | 0.118±0.052 | 0.203±0.054 |
| N (%) | 0.000±0.000 | 0.043±0.027 | 0.128±0.065 | 0.035±0.029 | 0.075±0.030 |
| Fe (mg/kg) | 819.315±93.579 | 860.328±47.390 | 790.795±87.203 * | 969.353±256.464 | 1464.085±178.420 |
| Cu (mg/kg) | 36.208±2.820 | 32.630±1.541 | 30.873±1.847 | 44.530±11.264 | 77.978±6.768 * |
| Zn (mg/kg) | 17.443±4.500 | 13.340±1.977 | 15.730±2.329 | 10.873±2.092 | 17.025±1.636 |
| Mn (mg/kg) | 36.843±3.811 | 38.693±2.646 | 38.450±3.399 | 41.575±8.177 | 58.895±5.766 |
| K (wt%) | 0.006±0.000 * | 0.007±0.000 * | 0.006±0.000 * | 0.003±0.000 | 0.003±0.000 |
| Na (wt%) | 0.017±0.003 | 0.019±0.001 | 0.018±0.001 | 0.014±0.000 | 0.015±0.000 |
| Mg (wt%) | 0.000±0.001 | 0.002±0.001 | 0.003±0.001 | 0.002±0.001 | 0.002±0.001 |
| Ca (wt%) | 0.216±0.032 | 0.244±0.025 | 0.250±0.023 | 0.186±0.029 | 0.254±0.016 |
| P (wt%) | 0.027±0.007 | 0.021±0.003 | 0.020±0.002 | 0.044±0.002 * | 0.048±0.002 * |
| S (wt%) | 0.013±0.005 | 0.004±0.001 | 0.004±0.002 | 0.000±0.000 | 0.007±0.011 |

* Significant at < 0.05 level, ** Significant at < 0.01 level

**Supplementary Table S5.** Soil bacteria two-way ANOVA results showing *P* values for alpha diversity calculators. Treatments consisted of ‘Fertiliser’ and 'Harvest'. Significant *P* values indicated by *, ** and *** corresponding to *P* < 0.05, *P* < 0.01 and < 0.001, respectively.

| Diversity index | Treatment | Degrees of freedom | Sum of Squares | Mean Squares | F. Model | *P* |
| --- | --- | --- | --- | --- | --- | --- |
| Inverse Simpson | Fertiliser | 4 | 11343.5 | 2835.9 | 3.098 | 0.030 * |
|  | Harvest | 1 | 3314.2 | 3314.2 | 3.620 | 0.067 |
|  | Fertiliser: Harvest | 4 | 10339.2 | 2584.8 | 2.824 | 0.042 * |
|  | Residuals | 30 | 27461.4 | 915.4 |  |  |
| Fisher | Fertiliser | 4 | 55610.0 | 13903.0 | 4.652 | 0.005 ** |
|  | Harvest | 1 | 36091.0 | 36091.0 | 12.076 | 0.002 ** |
|  | Fertiliser: Harvest | 4 | 67266.0 | 16816.0 | 5.627 | 0.002 ** |
|  | Residuals | 30 | 89657.0 | 2989.0 |  |  |
| Richness | Fertiliser | 4 | 1293488.0 | 323372.0 | 3.430 | 0.020 * |
|  | Harvest | 1 | 729540.0 | 729540.0 | 7.739 | 0.009 ** |
|  | Fertiliser: Harvest | 4 | 2351676.0 | 587919.0 | 6.237 | <0.001 *** |
|  | Residuals | 30 | 2828104.0 | 94270.0 |  |  |
| Evenness | Fertiliser | 4 | 0.004 | 0.0 | 6.328 | <0.001 *** |
|  | Harvest | 1 | 0.000 | 0.0 | 0.019 | 0.891 |
|  | Fertiliser: Harvest | 4 | 0.000 | 0.0 | 0.647 | 0.633 |
|  | Residuals | 30 | 0.004 | 0.0 |  |  |

**Supplementary Table S6.** Results of two-way ANOVA relative abundance of bacteria taxa. Treatments consisted of ‘Fertiliser’ and 'Harvest'. Significant *P* values indicated by *, ** and *** corresponding to *P* < 0.05, *P* < 0.01 and < 0.001, respectively.

| Taxon | Treatment | Degrees of freedom | Sum of Squares | Mean Squares | F. Model | *P* |
| --- | --- | --- | --- | --- | --- | --- |
| Actinobacteria | Fertiliser | 4 | 0.028 | 0.007 | 5.931 | 0.001** |
|  | Harvest | 1 | 0.008 | 0.008 | 6.618 | 0.015* |
|  | Fertiliser: Harvest | 4 | 0.013 | 0.003 | 2.708 | 0.049* |
|  | Residuals | 30 | 0.035 | 0.001 |  |  |
| Proteobacteria | Fertiliser | 4 | 0.001 | 0.000 | 0.563 | 0.692 |
|  | Harvest | 1 | 0.000 | 0.000 | 0.240 | 0.628 |
|  | Fertiliser: Harvest | 4 | 0.003 | 0.001 | 1.657 | 0.186 |
|  | Residuals | 30 | 0.012 | 0.000 |  |  |
| Acidobacteria | Fertiliser | 4 | 0.002 | 0.000 | 10.801 | < 0.001*** |
|  | Harvest | 1 | 0.000 | 0.000 | 9.699 | 0.004** |
|  | Fertiliser: Harvest | 4 | 0.000 | 0.000 | 1.228 | 0.320 |
|  | Residuals | 30 | 0.001 | 0.000 |  |  |
| Chloroflexi | Fertiliser | 4 | 0.000 | 0.000 | 2.814 | 0.043* |
|  | Harvest | 1 | 0.000 | 0.000 | 2.912 | 0.098 |
|  | Fertiliser: Harvest | 4 | 0.000 | 0.000 | 1.373 | 0.267 |
|  | Residuals | 30 | 0.001 | 0.000 |  |  |
| Firmicutes | Fertiliser | 4 | 0.004 | 0.001 | 32.007 | < 0.001*** |
|  | Harvest | 1 | 0.000 | 0.000 | 1.690 | 0.204 |
|  | Fertiliser: Harvest | 4 | 0.000 | 0.000 | 0.347 | 0.844 |
|  | Residuals | 30 | 0.001 | 0.000 |  |  |
| Gemmatimonadetes | Fertiliser | 4 | 0.000 | 0.000 | 3.071 | 0.031* |
|  | Harvest | 1 | 0.000 | 0.000 | 7.629 | 0.010** |
|  | Fertiliser: Harvest | 4 | 0.000 | 0.000 | 1.731 | 0.169 |
|  | Residuals | 30 | 0.001 | 0.000 |  |  |
| Planctomycetes | Fertiliser | 4 | 0.001 | 0.000 | 5.814 | 0.001** |
|  | Harvest | 1 | 0.000 | 0.000 | 11.915 | 0.002** |
|  | Fertiliser: Harvest | 4 | 0.000 | 0.000 | 1.725 | 0.171 |
|  | Residuals | 30 | 0.001 | 0.000 |  |  |

**Supplementary Table S7.** Soil bacterial community analysis by PERMANOVA results based on 97% similarity OTU abundance data (square root transformed), using 999 permutations. Treatments consisted of ‘Fertiliser’ and ‘Harvest’. Significant *P* values indicated by * and *** corresponding to P < 0.05 and < 0.001, respectively.

| Treatment | Degrees of freedom | Sum of Squares | Mean Squares | F. Model | R^2^ | *P* |
| --- | --- | --- | --- | --- | --- | --- |
| Fertiliser | 4 | 0.562 | 0.140 | 2.301 | 0.176 | 0.013* |
| Harvest | 1 | 0.158 | 0.158 | 2.587 | 0.049 | 0.043* |
| Fertiliser: Harvest | 4 | 0.642 | 0.160 | 2.629 | 0.201 | 0.003** |
| Residuals | 30 | 1.830 | 0.061 | 0.574 |  |  |
| Total | 39 | 3.192 | 1 |  |  |  |

**Supplementary Table S8.** ANOVA results of PICRUSt data for nitrogen cycling genes. Treatments consisted of ‘Fertiliser’, and 'Harvest'. Significant *P* values indicated by * and *** corresponding to P < 0.05 and < 0.001, respectively.

|  | Treatment | Degrees of freedom | Sum of Squares | Mean Squares | F. Model | Pr(>F) |
| --- | --- | --- | --- | --- | --- | --- |
| *nifD* | Fertiliser | 4 | 1075704 | 268926 | 2.111 | 0.104 |
|  | Harvest | 1 | 792141 | 792141 | 6.218 | 0.018* |
|  | Fertiliser: Harvest | 4 | 2625705 | 656426 | 5.153 | 0.003** |
|  | Residuals | 30 | 3821955 | 127398 |  |  |
| *amoA.amoB* | Fertiliser | 4 | 117.4 | 29.35 | 2.453 | 0.067 |
|  | Harvest | 1 | 36.1 | 36.1 | 3.017 | 0.093 |
|  | Fertiliser: Harvest | 4 | 31.4 | 7.85 | 0.656 | 0.627 |
|  | Residuals | 30 | 359 | 11.967 |  |  |
| *hao* | Fertiliser | 4 | 21779 | 5444.8 | 3.612 | 0.016* |
|  | Harvest | 1 | 10660 | 10660.2 | 7.071 | 0.012* |
|  | Fertiliser: Harvest | 4 | 23599 | 5899.9 | 3.914 | 0.011* |
|  | Residuals | 30 | 45226 | 1507.5 |  |  |
| *narG* | Fertiliser | 4 | 2339491 | 584873 | 3.490 | 0.019* |
|  | Harvest | 1 | 426629 | 426629 | 2.546 | 0.121 |
|  | Fertiliser: Harvest | 4 | 3274733 | 818683 | 4.885 | 0.004** |
|  | Residuals | 30 | 5027930 | 167598 |  |  |
| *nrfA* | Fertiliser | 4 | 9051.4 | 2262.8 | 6.469 | < 0.001*** |
|  | Harvest | 1 | 3724.9 | 3724.9 | 10.648 | 0.003** |
|  | Fertiliser: Harvest | 4 | 7772.3 | 1943.1 | 5.555 | 0.002** |
|  | Residuals | 30 | 10494.5 | 349.8 |  |  |
| *nirK* | Fertiliser | 4 | 565367 | 141342 | 3.352 | 0.022* |
|  | Harvest | 1 | 70224 | 70224 | 1.665 | 0.207 |
|  | Fertiliser: Harvest | 4 | 936981 | 234245 | 5.555 | 0.002** |
|  | Residuals | 30 | 1265171 | 42172 |  |  |
| *nosZ* | Fertiliser | 4 | 86867 | 21716.7 | 4.023 | 0.010** |
|  | Harvest | 1 | 1562 | 1562.5 | 0.289 | 0.595 |
|  | Fertiliser: Harvest | 4 | 126351 | 31587.8 | 5.851 | 0.001** |
|  | Residuals | 30 | 161964 | 5398.8 |  |  |

**Supplementary Table S9.** ANOVA results of PICRUSt data for carbon degrading genes. Treatments consisted of ‘Fertiliser’, and 'Harvest'. Significant *P* values indicated by * and *** corresponding to P < 0.05 and < 0.001, respectively.

|  | **Treatment** | **Degrees of freedom** | **Sum of Squares** | **Mean Squares** | **F. Model** | **Pr (>F)** |
| --- | --- | --- | --- | --- | --- | --- |
| alpha.amylase | Fertiliser | 4 | 406037 | 101509 | 4.343 | 0.007** |
|  | Harvest | 1 | 1918 | 1918 | 0.082 | 0.776 |
|  | Fertiliser: Harvest | 4 | 562406 | 140602 | 6.015 | 0.001** |
|  | Residuals | 30 | 701247 | 23375 |  |  |
| glucoamylase | Fertiliser | 4 | 637337 | 159334 | 2.746 | 0.047* |
|  | Harvest | 1 | 430770 | 430770 | 7.424 | 0.011* |
|  | Fertiliser: Harvest | 4 | 1278337 | 319584 | 5.508 | 0.002** |
|  | Residuals | 30 | 1740630 | 58021 |  |  |
| beta.galactosidase | Fertiliser | 4 | 5153311 | 1288328 | 2.473 | 0.066 |
|  | Harvest | 1 | 3125369 | 3125369 | 5.998 | 0.020* |
|  | Fertiliser: Harvest | 4 | 11786269 | 2946567 | 5.655 | 0.001** |
|  | Residuals | 30 | 15631528 | 521051 |  |  |
| endoglucanase1 | Fertiliser | 4 | 26709781 | 6677445 | 2.438 | 0.069 |
|  | Harvest | 1 | 14191957 | 14191957 | 5.182 | 0.030* |
|  | Fertiliser: Harvest | 4 | 55978785 | 13994696 | 5.110 | 0.003** |
|  | Residuals | 30 | 82157900 | 2738597 |  |  |
| betaglucosidase | Fertiliser | 4 | 13429346 | 3357337 | 2.724 | 0.048* |
|  | Harvest | 1 | 4637610 | 4637610 | 3.762 | 0.062 |
|  | Fertiliser: Harvest | 4 | 26229461 | 6557365 | 5.320 | 0.002** |
|  | Residuals | 30 | 36978738 | 1232625 |  |  |
| chitinase | Fertiliser | 4 | 7406000 | 1851500 | 2.794 | 0.044* |
|  | Harvest | 1 | 2570490 | 2570490 | 3.879 | 0.058 |
|  | Fertiliser: Harvest | 4 | 14095546 | 3523887 | 5.318 | 0.002** |
|  | Residuals | 30 | 19877856 | 662595 |  |  |
| catalase | Fertiliser | 4 | 11656628 | 2914157 | 2.981 | 0.035* |
|  | Harvest | 1 | 3534302 | 3534302 | 3.616 | 0.067 |
|  | Fertiliser: Harvest | 4 | 20854902 | 5213725 | 5.334 | 0.002** |
|  | Residuals | 30 | 29323099 | 977437 |  |  |
